# Machine Learning Approaches for the Estimation of Biological Aging: The Road Ahead for Population Studies

**DOI:** 10.3389/fmed.2019.00146

**Published:** 2019-07-03

**Authors:** Alessandro Gialluisi, Augusto Di Castelnuovo, Maria Benedetta Donati, Giovanni de Gaetano, Licia Iacoviello, the Moli-sani Study Investigators

**Affiliations:** ^1^Department of Epidemiology and Prevention, IRCCS NEUROMED, Pozzilli, Italy; ^2^Mediterranea Cardiocentro, Naples, Italy; ^3^Department of Medicine and Surgery, University of Insubria, Varese, Italy

**Keywords:** biological age, aging, blood, brain, big data, machine learning, neuroimaging, neurological diseases

## Abstract

In recent years, different machine learning algorithms have been developed for the estimation of Biological Age (BA), defined as the hypothetical underlying age of an organism. BA can be computed based on different circulating and non-circulating biomarkers. In this perspective, identifying biomarkers with a prominent influence on BA and developing reliable models for its estimation is of fundamental importance for monitoring healthy aging, and could provide new tools to screen health status and the risk of clinical events in the general population. Here, we briefly review the different machine learning (ML) approaches used for BA estimation, focusing on those methods with potential application to the Moli-sani study, a prospective population-based cohort study of 24,325 subjects (35–99 years). In particular, we discuss the potential of BA estimation based on blood biomarkers, which likely represents the easiest and most immediate way to compute organismal BA. Similarly, we describe ML methods for the estimation of brain age based on structural neuroimaging features. For each method, we discuss the relation with epidemiological variables (e.g., mortality), genetic and environmental factors, and common age-related diseases (e.g., Alzheimer disease), to examine the potential as aging biomarker in the general population. Finally, we hypothesize new approaches for BA estimation, both at the single organ and at the whole organism level. Overall, here we trace the road ahead in the Big Data era for our and other prospective general population cohorts, presenting ways to exploit the notable amount of data available nowadays.

## Introduction

By 2050, over 21% of the global population will be over 60 years of age (1), with an increase in age-related diseases burden. In this context, in the last years scientists have developed estimators of Biological Age (BA), i.e., the hypothetical underlying age of an organism, which can be computed through a number of circulating and non-circulating biomarkers (2, 3). Therefore, identifying the biomarkers that are representative of BA or have a prominent influence on this parameter, and developing reliable models for its estimation, is of fundamental importance for monitoring healthy aging, which includes different concepts like avoiding disease and disability, maintaining good cognitive and physical function, and remaining actively engaged in life activities (4) (see Supplementary Materials). BA biomarkers could provide new tools to screen this status and the risk of clinical events in the general population (5).

To this end, scientists have investigated different variables, some of which show a typical decline with increasing age, and are therefore considered suitable biomarkers of aging [reviewed in Cole et al. (6)]. Such aging biomarkers are mostly based on univariate or multivariate regression methods, where BA is a function of one or few bodily measures. These include instrumental parameters such as spirometry measures (7, 8) and heart rate variability (9), circulating blood biomarkers (10), and molecular genetics measures like telomere length (11), transcriptomics of peripheral blood cells (12, 13) and DNA methylation patterns (14, 15). Although it would be interesting to review these measures [as in Cole et al. (6)], this perspective paper is aimed at briefly reviewing recent supervised machine learning (ML) approaches for the estimation of BA in population-based studies. Supervised ML covers different algorithms which learn to identify patterns and relations among many input variables (features) in order to estimate as accurately and as robustly as possible one or more output variables (labels) [see Fabris et al. (16) and Supplementary Materials]. Here we will present the potential of supervised ML methods for the estimation of BA compared to the above mentioned “classical” methods, focusing on the most recent advances which allow computing a systemic (blood-based) and a brain-specific age. These showed the best performances in terms of accuracy and prediction of mortality risk, and can be more easily applied within large longitudinal population studies, thanks to the availability of blood test and brain imaging data, and of clinical events. We will analyse the potential of these algorithms in the Moli-sani study, a population-based cohort of 24,325 citizens (age≥35 years; 51.5% women) from the Molise region, Italy (17). This paper is aimed at pointing the way for our and other groups facing the complexity of large population cohorts characterized by a high number of epidemiological, biological, and medical variables.

## Blood-Based Biological Age

Common blood tests usually allow to check up the general health of subjects and possibly detect the first signs of disease. Moreover, specific markers often tend to increase (e.g., glucose) or decline (e.g., hemoglobin) as the age of an organism progresses (18). For these reasons, the estimation of BA based on sets of blood biomarkers has always been a hotspot of investigation in aging research (2, 10, 18–20).

Recently, an innovative and relatively accurate method based on deep learning has been proposed to estimate BA, using circulating biomarkers as input features and chronological age (CA) as label (2, 20). Deep learning represents a specific branch of ML, aimed at identifying patterns and models to explain relations among a number of variables in a big data scenario. One of the most prominent examples of deep learning techniques is represented by Deep Neural Networks, which recognize patterns in large amounts of data by imitating the architecture and functionality of the brain, in which we have an input layer, one or many hidden “decision” layers and an output layer (21). This way, for each vector of input features provided (i.e., the blood test of a given subject), the algorithm returns a predicted BA value (20). These algorithms are capable of capturing hidden underlying features and learning complex representations of highly multidimensional data (22).

### Properties and Characteristics of Biological Age

In the first pioneering study, Putin and colleagues (20) used anonymized blood biochemistry records from 62,419 subjects from the general Russian population to estimate BA through a ML approach, based on 41 standardized blood markers, age and sex of subjects. Deep Neural Networks showed the best performance in predicting BA, when compared to other algorithms, with a standard coefficient of determination (*R*^2^, the fraction of variance in CA explained by the model) of 0.8, a Pearson correlation coefficient between CA and BA (r) of 0.9, and a Mean Absolute Error (MAE, indicating the average disagreement between CA and BA) of 6.07 years. Based on this evidence, the authors tested 40 different networks on the same input data, with varying hyperparameters (such as number of layers and number of neurons in each layer), and finally built an ensemble of the 21 most predictive models. This raised the performance of the model to *R*^2^ = 0.83, *r* = 0.91, and MAE = 5.55 (Table 1), reaching accuracy values higher than blood transcriptomic biomarkers of aging [*R*^2^ = 0.6; (12)], but lower than epigenetic biomarkers previously published [Pearson *r* = 0.91 and 0.96, (14, 15); *R*^2^ = 0.93 and 0.89, (20)]. When a core set of the 10 most predictive circulating biomarkers—based on a Permutation Feature Importance analysis—were included in the networks, accuracy statistics were still good (*R*^2^ = 0.63), supporting a substantial robustness of the model (20).

**Table 1:** Main accuracy metrics of the biological age estimates described here.

**Tissue type**	**Input features**	**ML algorithm**	**N (training:test)**	**Population**	**Pearson r**	***R*^**2**^**	**MAE (years)**	**Reference**
Blood	41 haemochrome markers	DNN	62,419 (90:10)	Russian	0.9	0.8	6.07	(20)
	19 haemochrome markers	DNN	65,760 (80:20)	South Korean	0.7	0.49	5.59	(2)
			55,920 (80:20)	Eastern European	0.84	0.69	6.25	
			20,699 (80:20)	Canadian	0.7	0.52	6.36	
			142,379 (80:20)	All	0.8	0.65	5.94	
Brain	Structural MRI (normalized GM volumes)	GPR	2,001 (90:10)	different ancestries	0.95	0.89	4.66	(23)
	Structural MRI (normalized WM volumes)				0.92	0.84	5.88	
	Structural MRI (normalized GM + WM volumes)				0.96	0.91	4.41	
	Structural MRI (raw data)				0.57	0.32	11.81	
	Structural MRI (normalized GM volumes)	CNN			0.96	0.92	4.16	
	Structural MRI (normalized WM volumes)				0.94	0.88	5.14	
	Structural MRI (normalized GM + WM volumes)				0.96	0.91	4.34	
	Structural MRI (raw data)				0.94	0.88	4.65	
	Structural MRI (normalized GM + WM volumes)	GPR	2,001 (80:10:10)	different ancestries	0.94	0.88	5.02	(5)

*ML, Machine Learning; MAE, Mean Absolute Error; DNN, Deep Neural Networks; GPR, Gaussian Process Regressions; CNN, Convolutional Neural Networks; MRI, Magnetic Resonance Imaging; GM/WM, gray/white matter*.

Mamoshina et al. (2) exploited these models to train similar algorithms on population-specific datasets. In this work, samples from three ethnically different populations were used, including a South Korean (*N* = 65,760), an Eastern European (*N* = 55,920) and a Canadian dataset (*N* = 20,699). Networks were trained within each population and tested on independent test sets of all the populations available, based on sex and 19 circulating markers. Such models showed good predictive values across the three datasets, when they were trained and tested on the same population (*R*^2^ ranging from 0.49 to 0.69, and MAE ranging between 5.59 and 6.36 years). However, accuracy values dropped when the models trained in a given population were tested on a different ethnicity (*R*^2^ = [0.24; 0.34]; MAE = [7.1; 9.77] years). In line with this evidence, when networks were trained on a combination of the three datasets, including the population label as additional feature, this resulted in an increased accuracy, both when the model was tested on single populations (*R*^2^ = [0.49; 0.70]; MAE = [5.60; 6.22] years) and when tested on a combination of them (*R*^2^ = 0.65; MAE = 5.94). This evidence suggested a substantial population-specificity of these models (2), which may be due to different exposure of these populations to environmental factors or, to a lesser extent, to distinct genetic ancestry. A Permutation Feature Importance analysis detected five important features in age prediction, concordantly across the three populations: sex, albumin, glucose, hemoglobin and urea levels. Of note, women showed more accurate predictions than men (2), and variation in the levels of these circulating biomarkers was associated with physiological aging, as well as with age-related conditions (18).

More recently, Mamoshina et al. (24) reported the first evidence of a positive association between tobacco smoking and BA estimates, with smoking females and males being twice and one and a half times as old as their non-smoking counterparts, respectively. This significant difference was prominent under the age of 40 and held for all age ranges up to 55 years, after which the discrepancy disappeared, probably due to the increasing survival of subjects resilient to smoke effects after this age (24).

### Biological Age Predicts All-Cause Mortality

One of the most important characteristics of aging clocks is the ability to predict mortality (2). The deep hematological age (BA) described above was tested through a survival analysis in two general population cohorts, the Canadian dataset and the National Health and Nutrition Examination Survey (NHANES), from US (*N* = 2,768). For each sample, authors computed the difference between BA and CA (ΔBA = BA–CA), and carried out a Cox proportional hazards regression model on all-cause mortality events, adjusted for age and sex. They observed that subjects with slowed aging (ΔBA <-5) showed a decreased mortality risk compared to the normal group (−5 <= ΔBA <= 5) (from 30.4 to 24% in the NHANES and from 49.2 to 31.5% in the Canadian cohort). Accordingly, subjects with accelerated aging (ΔBA>5) had a higher mortality risk compared to the normal group, although Hazard Ratios were not always significant across all the trained models (2). This supported ΔBA as a robust marker of public health in the general population, suggesting it could be used to screen health status and mortality risk in populations, through the use of cheap and easy-to-obtain medical health records.

## Brain-Based Biological Age

An independent focus of investigation in the field of aging has pointed to the estimation of Brain Age (BrA), based on multi-modal brain imaging data [(5, 23, 25–30), reviewed in Cole et al. (6)]. Here, we will focus on the most recent and accurate developments, which use structural Magnetic Resonance Imaging (MRI) data (5, 6, 23, 27, 28, 31, 32). This method is based on Gaussian Process Regressions, an algorithm which, starting from an NxN similarity matrix of normalized gray and white matter images, computes a predicted BA value through a regression task, extending, and combining multivariate Gaussian distributions over a high number of features. This allows to reflect local patterns of covariance between individual data points (23).

### Properties and Characteristics of Brain Age

A recent methodological work reported BrA to be relatively accurate in predicting CA in a large dataset of healthy controls (*N* = 2,001), showing *R*^2^≥0.91 and MAE = 4.16 years (23). Convolutional Neural Networks—feed-forward neural networks in which the inputs are grouped spatially into hidden nodes (21)—had comparable results in terms of accuracy, which was preserved also when the model was fed with raw (non-parcelated) data (*R*^2^ = 0.88, MAE = 4.65 years). Interestingly, both methods reached the best performance when they were fed with both gray and white matter neuroimaging data (23). BrA estimates show high levels of within scanner and between-scanner reliability, with Intraclass Correlations Coefficients ≥0.92 for Gaussian Process Regressions and ≥0.85 for Convolutional Neural Networks, when applied to gray and white matter data together (23). Moreover, BrA is moderately heritable, after correcting for CA (h_2_ ≥ 0.5) (23). Although this preliminary finding is based on a small sample of monozygotic and dizygotic twins (*N* = 62) and needs further support from larger analyses, it suggests that genetic factors may have an important influence on BrA (33), as already reported for a number of structural and functional brain measures [reviewed in Jansen et al. (34)].

### Environmental Effects on Brain Age

Environmental exposures such as diet, physical activity, and educational attainment have been positively associated with important component measures of BrA, like cortical thickness (35–37), total brain, gray and white matter volume (38, 39). In line with this evidence, years of education and self-reported physical activity were found to be positively associated with the discrepancy between BrA and CA (hereafter called ΔBrA) in healthy adults, where BrA was based on cortical/subcortical gray matter measures (40). Of note, these associations were all observed in cross-sectional studies, underlining the need for longitudinal analyses to identify causal trajectories. Similarly, cross-sectional studies reported a positive association of younger BrA with meditation activity (−7.5 years) (41), and with long-term amateur music-playing (−4.03 years) (42).

Interestingly, the effect of dietary patterns on BrA has been also investigated, but only in baboons: a premature brain aging (+2.7 years) was observed in young female adults who had experienced moderate fetal undernutrition, compared to healthy controls (43). More recently, Hatton and colleagues (44) reported an association between negative fateful life events and advanced brain aging (+2.3 years), after controlling for physical, psychological, and lifestyle factors.

### Brain Age in Health Conditions

A number of structural MRI measures which are considered as proxies of brain aging –such as cortical thickness and white matter integrity- have been used to test associations with several health conditions, including obesity, diabetes, mild cognitive impairment, and Alzheimer disease. However, only a few works have explicitly tested association of BrA with such disorders [see Cole and Franke (3) for a comprehensive review]. Studies reported an accelerated brain aging for subjects affected by HIV (+2.2 years) (32), Down Syndrome (+2.5 years) (28) and medically refractory focal epilepsy (+4.5 years) (45), as well as for subjects who had previously experienced traumatic brain injury (+4.66 and +5.79 years for gray- and white matter-based models, respectively) (27) (Table 2).

**Table 2:** Associations of biological age estimates described here with health conditions, mortality risk, frailty measures, and environmental factors.

**Tissue type**	**Mortality risk: Test Cohort, N, HR**	**Reference**	**Health conditions (Δage, years)**	**Reference**	**Healthy aging measures (β, p)^a^**	**Reference**	**Environmental influences (AR/Δage/β)^b^**	**Reference**
Blood	NHANES (US), *N* = 2,768, HR = 1.71	(2)	None reported	NA	None reported	NA	Tobacco smoking (Age <30: AR ~1.62; Age 31–40: AR ~1.32; Age 41–50: AR ~1.15)	(24)
	Canada, N = 20,699, HR = 1.66							
Brain	LBC1936 (Scotland), N = 669, HR = 1.06	(5)	HIV (+2.2)	(32)	Fluid cognitive performance (β = −0.12, p = 0.007)	(5)	Meditation (Δage = −7.5 years)	(41)
			Down Syndrome (+2.5)	(28)	Grip strength (β = −0.06, p = 0.020)		Physical activity (β = −0.58 years per flights of stairs climbed)	(40)
			Focal Epilepsy (medically refractory: +4.5; newly diagnosed: +0.9)		Lung function (β = −0.072, *p* = 0.020)		Education (β = −0.95 yrs per school year)	
				(45)			Music playing (amateur: Δage = −4.03; professional: Δage = −3.22 years)	(42)
							Malnutrition (females: Δage = +2.74; males: Δage = +0.03)	(43)
			TBI (GM: +4.66; WM: +5.79)	(27)	Walking speed (β = 0.13, p = 0.004)		Negative life events (β = +0.37 yrs per event)	(44)

*NHANES, National Health and Nutrition Examination Survey; LBC1936, Lothian Birth Cohort 1936; HIV, Human Immunodeficiency Virus; TBI, traumatic brain injury; HR, Hazard Ratio; ΔAge, difference between biological and chronological age; AR, aging ratio between biological and chronological age*.

a *Healthy aging (frailty) measures: cognitive function, fluid-type intelligence; grip strength, right-hand grip strength (measured by a dynamometer); lung function, forced expiratory volume in 1 second (FEV_1_); walking speed, time to walk 6 meters. Beta and p-value of linear association between Δage and frailty measures are reported*.

b *Δage (years) are reported where available, unless otherwise stated. For blood-based BA, we report inferred Aging Ratios (24) up to the age of 50, after which the ratio reaches a plateau. For physical activity, education and negative life events, we report the resulting change in Δage per unitary increase of the independent variable*.

Much of the research on health conditions focused on disorders and physiological processes strictly related with aging, such as cognitive decline, Alzheimer disease and cognitive impairment. Along with direct associations with Alzheimer disease [+10 years, (26); +6.7 years, (30)], associations with progressive cognitive impairment [+6.2 years; (46)] and with cognitive decline in traumatic brain injury (27) and HIV patients (32) have been reported. Interestingly, also links with biomarkers of dementia were detected: in Down Syndrome patients, ΔBrA was associated with cerebral beta amyloid deposition -which represents a critical event in Alzheimer pathology- in addition to cognitive performance (28). Similarly, in progressive cognitive impaired and Alzheimer subjects, carriers of the *APOE* ε*4* allele—coding for a specific isoform of apolipoprotein E which increases the risk of late onset dementia—showed a sharper increase of ΔBrA along a 3-years follow-up, compared to non-carriers (47). In spite of these findings, BrA has been scarcely investigated in relation to cognitive functions in the general population, with a single association with lower fluid intelligence reported (5).

Of note, in longitudinal studies, subjects affected by some of the conditions mentioned above showed an increased discrepancy between BrA and CA along the observation period. As an example, Alzheimer cases showed an increase of ΔBrA of 2.3 years after 2 years of follow up (30).

Overall, these findings are in line with evidence that age and disease share common biological mechanisms (48), in addition to neuro-anatomical signs (33).

### Brain Age Is Associated With Frailty and Predicts Mortality

As with the discrepancy between (blood-based) BA and CA (ΔBA), also the difference with BrA (ΔBrA) warrants further validation as a public health marker. In a recent study on the general British population (*N* = 669), Cole et al. (5) supported this hypothesis, reporting a 6.1% increase in the relative risk of all-cause mortality between the age of 72 and 80, for each year of increase in ΔBrA (after correction for age and sex). This effect remained substantially unaffected when the analysis was further adjusted for additional variables related with mortality, including IQ, paternal social class, years of education, *APOE* ε*4* carrier status, smoking status and self-reported hypertension/diabetes/cardiovascular disease (5.1% risk increase) (5). Interestingly, ΔBrA explained more variance than long-established markers of BA like global DNA methylation and telomere length, and represented an independent survival predictor from these genetic biomarkers of aging. Prominently, when BrA was combined with epigenetic age (15), the global predictor showed an improved performance over all previous BA estimates (5), suggesting that combining estimators based on different biomedical sources may help improve mortality markers in the general population.

In the same study, authors investigated also the relation of ΔBrA with frailty, observing significant associations with weaker grip strength, poorer lung function, slower walking speed, and lower fluid intelligence (Table 2). Moreover, they observed a positive association with allostatic load, a composite measure of physiological and biological parameters which reflects the accumulation of “wear and tear” signs during lifespan (5).

## Future Perspectives

The discoveries reviewed above support the use of age discrepancies based on blood tests and neuroimaging data as sensible markers of public health in the general population.

However, these algorithms still present some limitations. First, the “black box” effect associated to many ML algorithms often does not allow to completely understand the relationships among features and labels and how they are estimated. Classical statistical methods and the knowledge of the medical/biological problem are of great help in this case. Second, the difference between BA and CA is basically a measure of prediction error of CA, which includes not only the discrepancy due to actual biological aging, but also errors associated to the input parameters used. This extends to any instrumental, biochemical or biometric measure potentially used as input feature. Moreover, these aging markers may be further improved at the methodological level and require validation in independent populations (2, 6). To reach this goal, in the present paper we would like to draw a strategy to further develop and test such markers in our population-based cohort, the Moli-sani study. A summary of all the variables and observations available in our cohort is reported in Table S1.

Thanks to the availability of instrumental measures, additional organ- or system-specific BA estimates could be developed and tested in our cohort (Figure S1). As an example, spirometry variables could help us compute a lung age which better predicts the pulmonary function of subjects, compared to models developed so far, which exploited one (7) or few variables (8). Similarly, we could exploit instrumental measures from electrocardiogram to compute a heart age, or better try to include blood- and vessels-related variables to build a more comprehensive model which predicts BA of the cardiovascular system. Another ambitious project consists of developing a comprehensive model for the estimation of BA, based on all the features available at baseline, and test the predictivity of this “holistic BA” in terms of mortality, frailty and other variables of public health interest (e.g., hospitalizations). Given the availability of DNA samples in our biobank, these approaches could possibly include also genetic and epigenetic data. This would allow us to implement new models for the estimation of BA, which may be more accurate and robust, in light of the complementarity of epigenetic clocks with independent BA measures (5), of their strong correlation with CA (14, 15), and of the number of genetic associations already detected with longevity (49). To the best of our knowledge, the above mentioned approaches have never been attempted before, hence they would represent a notable improvement compared to models currently available.

Overall, the novel scenarios opened up by the availability of massive volumes of data in health research and by the possibility to link them with biological and environmental variables from different sources will hopefully allow us to take part to the “Big Data revolution.”

## Author Contributions

AG wrote the first draft of the manuscript, with contributions and revisions from all the co-authors. All the authors accepted the final submitted version.

### Conflict of Interest Statement

The authors declare that the research was conducted in the absence of any commercial or financial relationships that could be construed as a potential conflict of interest.
