# Supplementary material for: Machine Learning Approaches for the Estimation of Biological Aging: The Road Ahead for Population Studies
Source: Front Med (Lausanne). 2019 Jul 3;6:146. doi: 10.3389/fmed.2019.00146 (PMC6626911; doi:10.3389/fmed.2019.00146)
Supplement: Supplementary file 1 [file Data_Sheet_1.pdf]

*Supplementary Material*

# Machine Learning Approaches for the Estimation of Biological Ageing: the Road Ahead for Population Studies

Alessandro Gialluisi, Augusto Di Castelnuovo, Maria Benedetta Donati, Giovanni de Gaetano and Licia Iacoviello, on behalf of the Moli-sani Study Investigators

**Correspondence:** Alessandro Gialluisi: alessandro.gialluisi@moli-sani.org

## The Moli-sani Study Investigators

**Steering Committee:** Licia Iacoviello*°(Chairperson), Giovanni de Gaetano* and Maria Benedetta Donati*.

**Scientific secretariat:** Licia Iacoviello*° (Coordinator), Marialaura Bonaccio*, Americo Bonanni*, Chiara Cerletti*, Simona Costanzo*, Amalia De Curtis*, Giovanni de Gaetano*, Augusto Di Castelnuovo*, Maria Benedetta Donati*, Francesco Gianfagna*°, Mariarosaria Persichillo*, Teresa Di Prospero* (Secretary).

**Safety and Ethycal Committee:** Jos Vermylen (Catholic Univesity, Leuven, Belgio) (Chairperson), Ignacio De Paula Carrasco (Accademia Pontificia Pro Vita, Roma, Italy), Simona Giampaoli (Istituto Superiore di Sanità, Roma, Italy), Antonio Spagnuolo (Catholic University, Roma, Italy).

**External Event adjudicating Committee**: Deodato Assanelli (Brescia, Italy), Vincenzo Centritto (Campobasso, Italy).

**Baseline and Follow-up data management:** Simona Costanzo* (Coordinator), Marco Olivieri (Università del Molise, Campobasso, Italy).

**Informatics:** Marco Olivieri (Università del Molise, Campobasso, Italy).

**Data Analysis:** Augusto Di Castelnuovo* (Coordinator), Marialaura Bonaccio*, Simona Costanzo*, Alessandro Gialluisi*, Francesco Gianfagna*°, Emilia Ruggiero*.

**Biobank and biomedical analyses:** Amalia De Curtis* (Coordinator), Sara Magnacca*.

**Genetic analyses:** Benedetta Izzi* (Coordinator), Francesco Gianfagna*°, Claudio Grippi*, Annalisa Marotta*, Fabrizia Noro*.

**Communication and Press Office:** Americo Bonanni* (Coordinator), Francesca De Lucia (Associazione Cuore Sano, Campobasso, Italy).

**Recruitment staff:** Mariarosaria Persichillo* (Coordinator), Francesca Bracone*, Francesca De Lucia (Associazione Cuore Sano, Campobasso, Italy), Salvatore Dudiez*, Livia Rago*.

**Follow-up Event adjudication:** Livia Rago* (Coordinator), Simona Costanzo*, Amalia De Curtis*, Licia Iacoviello*°, Teresa Panzera*, Mariarosaria Persichillo*.

**Regional Health Institutions:** Direzione Generale per la Salute - Regione Molise; Azienda Sanitaria Regionale del Molise (ASReM, Italy); Molise Dati Spa (Campobasso, Italy); Offices of vital statistics of the Molise region.

**Hospitals:** Presidi Ospedalieri ASReM: Ospedale A. Cardarelli – Campobasso, Ospedale F. Veneziale – Isernia, Ospedale San Timoteo - Termoli (CB), Ospedale Ss. Rosario - Venafro (IS), Ospedale Vietri – Larino (CB), Ospedale San Francesco Caracciolo - Agnone (IS); Casa di Cura Villa Maria - Campobasso; Fondazione di Ricerca e Cura Giovanni Paolo II - Campobasso; IRCCS Neuromed - Pozzilli (IS).

* Department of Epidemiology and Prevention, IRCCS Neuromed, Pozzilli, Italy

°Department of Medicine and Surgery, University of Insubria, Varese, Italy

*Baseline Recruitment staff is available at*

*http://www.moli-sani.org/index.php?option=com_content&task=view&id=21128&Itemid=118*

The enrolment phase of the Moli-sani Study was conducted at the Research Laboratories of the Catholic University in Campobasso (Italy), the follow up of the Moli-sani cohort is being conducted at the Department of Epidemiology and Prevention of the IRCCS Neuromed, Pozzilli, Italy.

## Healthy ageing: definition and measures

Although it is difficult to define healthy (or successful) ageing in a precise manner, this can be resumed as a composite condition characterized by lack of disease or illness, maintenance of intact physical and cognitive functions and active engagement in every-day life activities (1,2). This definition implies that different domains should be assessed to measure the healthy ageing status, including physiological and metabolic health, physical capability, cognitive function, psychological and social wellbeing (3). As a consequence, a number of measures have been proposed to investigate these domains (3,4). In addition to morbidity and mortality rates, some of the most used measures include instrumental measures of frailty, such as walking speed (time spent to walk a given distance), strength of hand-grip, cognitive performance and fluid intelligence (measured through specific psychometric tests) or lung function, which can be tested through different instrumental parameters (e.g. forced expiratory volume in 1 second, also known as $FEV_1$) (3,4). However, these measures work well in the elder population, while their applicability and efficacy among younger people is doubtful (4). This limitation partially applies also to measures of psychosocial wellbeing, which are aimed at assessing domains like depression and quality of life, often affected in the elders. Other complementary measures include more objective parameters, like cardiovascular parameters and hospitalization events, although much work remains to be done in order to validate these as healthy ageing indexes (4). In all likelihood, one of the most effective approaches to measure healthy ageing remain to test all of the relevant domains through specific tests and/or parameters, and then build composite indexes, as already proposed elsewhere (3).

## Supervised machine learning: a brief definition

Supervised machine learning represents a group of algorithms which, based on a number of input variables (or *features*), learn to predict a known outcome (either categorical or continuous variables), usually called *label*. This is accomplished through a phase in which the algorithm trains to predict the label as accurately as possible, which takes place in a *training set*, and a phase where the accuracy and robustness of the model is tested in an independent dataset, the *test set*. The advantage of ML algorithms is that they allow to model complex relationships of several features with the label, in a way that could not be possible through classical statistical methods.

*Accuracy metrics*

The following parameters are used as accuracy metrics for supervised ML algorithms aimed at the estimation of Biological Age (5).

*Pearson correlation coefficient*, which shows the strength of linear association between Chronological Age (CA) and Biological Age (BA)

$$r = \frac{\sum_{i=1}^{N}(x_i - \bar{x})(y_i - \bar{y})}{\sqrt{\sum_{i=1}^{N}(x_i - \bar{x})^2}\sqrt{\sum_{i=1}^{N}(y_i - \bar{y})^2}},$$

where $x_i$ is the CA value and $x^{-}$ is the mean of $x$, $y_i$ is the predicted BA value and $y^{-}$ is the mean of $y$, $N$ is the number of samples.

*Coefficient of determination*, indicating the proportion of variance in CA explained by BA

$$R^2 = 1 - \frac{\sum_{i=1}^{N}(\hat{y}_i - y_i)^2}{\sum_{i=1}^{N}(y_i - y')^2},$$

where $y_i$ is the real CA value, $y_i$^ is the predicted BA value, and $y'$ is the mean of $y$.

*Mean absolute error*, which represents the average discrepancy (in absolute value) between Biological and Chronological Age

$$MAE = \frac{1}{N}\sum_{i=1}^{N}|\hat{y}_i - y_i|,$$

where $y_i$^ is the predicted BA value, $y_i$ is is the real CA value, and $N$ is a number of samples over which the average is computed.

Given an arbitrary epsilon (ε) value, *ε-accuracy* represents the probability that a given predicted BA value falls within a ± ε years interval from the CA value. In other words,

$$\varepsilon - accuracy = \frac{\sum_{i=1}^{N} 1_A(\hat{y}_i)}{N},$$

where $A = [y_i - \varepsilon; y_i + \varepsilon]$, $y_i$ is the real CA value, $y_i$^ is the predicted BA value. This measure is of course influenced by the $\varepsilon$ value chosen, which determines the width of the interval and the level of accuracy of the statistics. As an example, for a subject with CA = 60 years and a predicted BA = 54 years, the ML model is considered to predict correctly BA if ε = 10 (i.e., within the age range [50;70] years), but not if ε = 5 (i.e., within the age range [55;60]).

Given a Biological and Chronological Age value, *log2 Aging Ratio* represents an index of the relationship between the two measures (i.e. how larger or smaller BA is compared to CA in a subject):

$$log_2 Aging\ ratio = log_2\left(\frac{\hat{y}_i}{y_i}\right),$$

where $y_i$^ is the predicted BA value and $y_i$ is is the real CA value. A *log2 Aging ratio* of 1 indicates that the sample is predicted to be twofold older than its CA, while a log2 Aging ratio of −1 means the sample is predicted to be half as old.

**Brief overview of variables and data available in the Moli-sani study**

The Moli-sani study is a population-based cohort of 24,325 citizens (age≥35 years; 51.5% women) from the Molise region, Italy, recruited between 2005 and 2010. In this cohort - which represents about 10% of the total Molise population – a number of clinical, biochemical, lifestyle, instrumental and other medical variables of interest have been collected, with the purpose of investigating genetic and environmental risk/protection factors for different clinical conditions (14). These include:

- anthropometric measures, personal and family history of health and disease;

- the Italian version of the EPIC food frequency questionnaire (15), allowing analyses of diet and dietary components;
- instrumental spirometry and electrocardiogram (ECG) measures;
- blood circulating biomarkers, including basic biochemical tests, blood cell counts, and many others;
- socio-economic variables, including educational level, household income, occupational class, housing, socioeconomic status during childhood, marital status, household crowding;
- psychometric scores including health-related quality of life, psychological resilience, depression and anxiety and suicidal ideation.

In addition, we have carried out passive follow-up based on linkage with hospital discharge records and regional mortality registry- at December 2011 (median follow-up time 4.3 years) and at December 2014 (7.5 years). Outcomes analysed included mortality for all and specific causes, hospitalizations, coronary artery disease, stroke, atrial fibrillation, heart failure, diabetes and cancer. We are currently starting a project to link these data with Magnetic Resonance Imaging (MRI), Positron Emission Tomography (PET) and other neuroimaging analyses carried out in our household clinics (IRCCS Neuromed). Moreover, we have the potential to retrieve drug therapy information for each of our subjects, thanks to the Drug prescription registry of the Regional Health System (ASReM), and to rescue information about exposure to environmental pollution through geo-localization and linkage to detailed particulate matter (PM) levels maps (16).

In 2018, the active follow-up recruitment of the cohort was started, to re-run all the tests previously administered at baseline, as well as new cognitive tests, and we are planning to carry out  MRI scanning of part of the subjects involved. This will allow us to exploit further longitudinal data to improve our predictions and models.

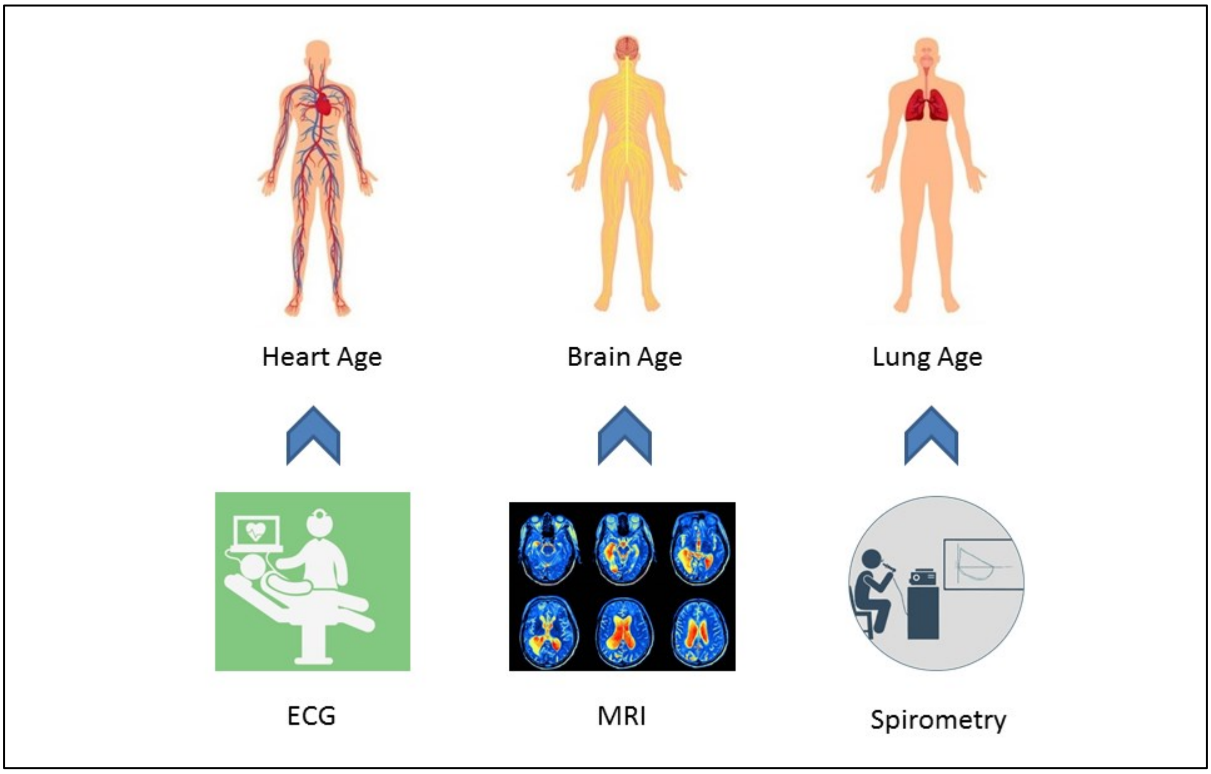


**Figure S1.** Different organ- and system-specific BA estimations, and potential sources of data available in the Moli-sani study. Here, we did not include blood-based methods, which we consider an approach for the estimation of organismal BA. Abbreviations: ECG = electrocardiogram; MRI = magnetic resonance imaging.

| Source | Variables # | Observations # |
|---|---|---|
| Diet Questionnaires | 1,600 | 38,920,000 |
| Spirometry | 153 | 3,721,725 |
| ECG | 617 | 15,008,525 |
| Clinical history | 2,100 | 51,082,500 |
| Family history of disease | 841 | 20,457,325 |
| Circulating biomarkers | 592 | 14,400,400 |
| Passive follow-up | 680 | 16,564,664 |
| Total | 6,583 | 160,155,139 |

**Table S1.** Summary of all the variables and observations available in the Moli-sani study for use in big data projects. Note: these figures do not include data produced by the active follow-up, which is currently ongoing, as well as potentially available data on drug prescriptions and environmental pollution (see above for details). Abbreviations: ECG = electrocardiogram.

**References**


1. Jin K. New perspectives on healthy aging. *Prog Neurobiol* (2017) **157**:1. doi:10.1016/j.pneurobio.2017.08.006

2. Cosco TD, Howse K, Brayne C. Healthy ageing, resilience and wellbeing. *Epidemiol Psychiatr Sci* (2017) **26**:579–583. doi:10.1017/s2045796017000324

3. Lara J, Godfrey A, Evans E, Heaven B, Brown LJE, Barron E, Rochester L, Meyer TD, Mathers JC. Towards measurement of the Healthy Ageing Phenotype in lifestyle-based intervention studies. *Maturitas* (2013) **76**:189–199. doi:10.1016/j.maturitas.2013.07.007

4. Mount S, Lara J, Schols AMWJ, Mathers JC. Towards a multidimensional healthy ageing phenotype. *Curr Opin Clin Nutr Metab Care* (2016) **19**:418–426. doi:10.1097/MCO.0000000000000318

5. Mamoshina P, Kochetov K, Putin E, Cortese F, Aliper A, Lee WS, Ahn SM, Uhn L, Skjodt N, Kovalchuk O, et al. Population specific biomarkers of human aging: a big data study using South Korean, Canadian and Eastern European patient populations. *J Gerontol A Biol Sci Med Sci* (2018) doi:10.1093/gerona/gly005

6. Putin E, Mamoshina P, Aliper A, Korzinkin M, Moskalev A. Deep biomarkers of human aging : Application of deep neural networks to biomarker development. (2016) **8**:1–13. doi:10.18632/aging.100968

7. Mamoshina P, Kochetov K, Putin E, Cortese F, Aliper A, Lee W-S, Ahn S-M, Uhn L, Skjodt N, Kovalchuk O, et al. Population specific biomarkers of human aging: a big data study using South Korean, Canadian and Eastern European patient populations. *Journals Gerontol Ser A* (2018) **00**:1–9. doi:10.1093/gerona/gly005

8. Cole JH, Poudel RPK, Tsagkrasoulis D, Caan MWA, Steves C, Spector TD, Montana G. Predicting brain age with deep learning from raw imaging data results in a reliable and heritable biomarker. *Neuroimage* (2017) **163**:115–124. doi:10.1016/j.neuroimage.2017.07.059

9. Cole JH, Ritchie SJ, Bastin ME, Valdés Hernández MC, Muñoz Maniega S, Royle N, Corley J, Pattie A, Harris SE, Zhang Q, et al. Brain age predicts mortality. *Mol Psychiatry* (2018) **23**:1385–1392. doi:10.1038/mp.2017.62

10. Cole JH, Underwood J, Caan MWA, De Francesco D, Van Zoest RA, Leech R, Wit

FWNM, Portegies P, Geurtsen GJ, Schmand BA, et al. Increased brain-predicted aging in treated HIV disease. *Neurology* (2017) **88**:1349–1357. doi:10.1212/WNL.0000000000003790

11. Cole JH, Annus T, Wilson LR, Remtulla R, Hong YT, Fryer TD, Acosta-Cabronero J, Cardenas-Blanco A, Smith R, Menon DK, et al. Brain-predicted age in Down syndrome is associated with beta amyloid deposition and cognitive decline. *Neurobiol Aging* (2017) **56**:41–49. doi:10.1016/j.neurobiolaging.2017.04.006

12. Pardoe HR, Cole JH, Blackmon K, Thesen T, Kuzniecky R. Structural brain changes in medically refractory focal epilepsy resemble premature brain aging. *Epilepsy Res* (2017) **133**:28–32. doi:10.1016/j.eplepsyres.2017.03.007

13. Cole JH, Leech R, Sharp DJ. Prediction of brain age suggests accelerated atrophy after traumatic brain injury. *Ann Neurol* (2015) **77**:571–581. doi:10.1002/ana.24367

14. Iacoviello L, Bonanni A, Costanzo S, Curtis A De, Castelnuovo A Di, Olivieri M, Zito F, Donati MB, Gaetano G de, Investigators TMP. The Moli-Sani Project, a randomized, prospective cohort study in the Molise region in Italy; design, rationale and objectives. *Ital J Public Health* (2007) **4**: doi:10.2427/5886

15. Pisani P, Faggiano F, Krogh V, Palli D, Vineis P, Berrino F. Relative validity and reproducibility of a food frequency dietary questionnaire for use in the Italian EPIC centres. *Int J Epidemiol* (1997) **26 Suppl 1**:S152-60. Available at: http://www.ncbi.nlm.nih.gov/pubmed/9126543 [Accessed September 8, 2018]

16. Stafoggia M, Schwartz J, Badaloni C, Bellander T, Alessandrini E, Cattani G, de' Donato F, Gaeta A, Leone G, Lyapustin A, et al. Estimation of daily PM10 concentrations in Italy (2006–2012) using finely resolved satellite data, land use variables and meteorology. *Environ Int* (2017) **99**:234–244. doi:10.1016/j.envint.2016.11.024
